# *Neospora caninum* Infection during the First Gestation of Holstein Heifers That Consume Food Contaminated Naturally with Zearalenone under Field Conditions

**Published:** 2017

**Authors:** Carlos CRUZ-VÁZQUEZ, Juan VITAL-GUTIÉRREZ, Leticia MEDINA-ESPARZA, Luis ORTEGA-MORA, Arturo VALDIVIA-FLORES, Teódulo QUEZADA-TRISTÁN, Agustín ORIHUELA-TRUJILLO

**Affiliations:** 1. Technological Institute El Llano Aguascalientes, Aguascalientes, Mexico; 2. Faculty of Veterinary, Complutense University of Madrid, Madrid, Spain; 3. Center of Agricultural Sciences, Autonomous University of Aguascalientes, Aguascalientes, México; 4. Faculty of Agricultural Sciences, Autonomous University of the State of Morelos, Cuernavaca, Morelos, México

**Keywords:** *
Neospora caninum
*, Natural infection, Zearalenone, Estradiol, Progesterone, Dairy cattle

## Abstract

**Background:**

This work studied the natural infection of *Neospora caninum* during the first gestation of heifers in a dairy farm in animals consuming a ration co ntaminated naturally with Zearalenone (ZEA), and to find out effect of mycotoxin in the levels of estrogen (E) and progesterone (P4) and that‘s relation to the infection to *N. caninum* and in the abortions.

**Methods:**

The study was conducted in a dairy farm located in El Llano municipality, in Aguascalientes, Mexico, in 2013. Two groups were formed, the group “A” with 20 seronegative animals to *N. caninum*, and group “B” with 20 seropositive. Once a month was determined the levels of total IgG to *N. caninum,* the serum concentration of E and P4, and the level of ZEA in the ration; in cases of abortion, fetal brain samples were taken to identify the presence of *N. caninum* DNA.

**Results:**

In group “A”, was observed two subgroups: seronegative (60%) and seroconverted (40%), and three abortions. In group “B”, all animals maintain their serostatus, and three animals aborted. All abortions were positive for *N. caninum* DNA. The level of ZEA in the ration has an average of 426 μg/kg; during the gestation did not identify that animals suffer any alteration in the levels of E or P4. No statistical differences among the studied variables (levels of E and P) in time (nine months of gestation) were detected. It was not identified any interaction with the natural exposure to ZEA intake in any of the groups under study.

**Conclusion:**

The chronic ingestion of ZEA does not affect serum concentrations of E and P4 during gestation of heifers under study and cannot be related to the infection for *N. caninum* and the abortion.

## Introduction

*Neospora caninum* (Apicomplexa, Sarcocystidae), is a protozoan parasite with heteroxene life cycle where the domestic dog and coyote appear as the main definitive hosts, while cattle and a wide range of animals, both domestic and wild, can act as intermediate hosts; herbivores can become infected by ingesting water and food contaminated with oocysts excreted by the definitive host, while a chronically infected female can transmit the parasite to the fetus through the placenta highly efficient way, it is responsible for the perpetuation of infection in herds (1, 2). In dairy cattle can cause early fetal death, embryo resorption, neonatal death, and abortions, especially in the second trimester of pregnancy, thus affecting the reproductive parameters, the replacement program and milk production (3).

Bovine neosporosis is recognized as a major cause of abortion in dairy cattle worldwide, estimated annual losses of $ 842.9 million dollars (4). In Mexico, the presence of *N. caninum* has been documented in cattle (5–7), sheep (8), as well as dogs (9, 10), and some wild species (11, 12), considered a major reproductive disease that causes losses of 68.5 and 94.8 million dollars annually in the dairy and meat industry, respectively (4).

Several risk factors to bovine neosporosis have been identified (1, 13); the literature reports poor feed quality and feeds contamination with mycotoxins as risk factors that may induce recrudescence of a latent *N. caninum* infection because mycotoxins cause immune suppression and reproductive disorders (14, 15).

Zearalenone (ZEA) is a mycotoxin produced by genus *Fusarium*, found as a natural contaminant of corn, barley, wheat, oats, sorghum and silage of these cereals, particularly in corn and wheat. ZEA is an estrogenic nonsteroidal mycotoxin whose toxic effects seems to depend on their interaction with estrogen receptors and are associated with infertility, enlargement of the mammary gland, reduced milk production, and vaginitis, especially in heifers, also has been reported hyperestrogenism, rectal and vaginal prolapse as well as abortions, delayed ovulation, flaws in the conception, implantation and development of the fetus (16, 17). There is no information in the literature on the effect of the ZEA in animals infected with *N. caninum* under field conditions, information needed to confirm its role in the epidemiology of this parasitic disease.

This work was developed under field conditions to study the dynamics of the natural infection of *N. caninum* during the first gestation of Holstein heifers in a dairy farm in animals consuming a ration contaminated naturally with ZEA. Moreover, to find out if this mycotoxin has some effect on the levels of estrogen and progesterone in the animals under study and if this effect could have some relation with the infection for *N. caninum* and eventually in the abortions.

## Materials and Methods

### Study site

This observational study, conducted on a dairy farm located in the El Llano municipality, in the state of Aguascalientes, located in the north-central Mexico, at an average altitude of 1995 m above sea level, with a semi-dry warm climate with summer rains. The farm maintains Holstein cattle in a free confinement system, characterized by open pens, with dirt floors, shaded area, and concrete drinking and feeding troughs, providing 40 m^2^ per animal (18). The ration consisted of silage triticale (58%), corn silage (38%), triticale as hay milled (3%), and a commercial concentrate with 14% crude protein (1%), the ingredients were properly mixed to offer themselves as totally mixed ration (TMR), supplied twice daily (7:00 and 15:00). The dairy was free of brucellosis and had a vaccination program to prevent BVD, IBR and leptospirosis. The farm had the presence of five dogs of different breed, sex and age, circulating freely in the facilities, which eventually consumed aborted fetuses and fetal residues.

### Groups under study

Two groups were formed, each with 20 heifers with 12 to 14 months of age, divided as follows: Group A, with 20 seronegative animals to *N. caninum*, and Group B, with 20 seropositive. A serum sample from each heifer was subjected to indirect ELISA test as described later (19) to determine his serological status before to start the study. All animals were individually identified and maintained in the same lodging pen. The heifers were artificially inseminated at estrous naturally present and were monitored since its first service until presents calving or abortion. Information about reproductive management and health were registered.

### Sampling

Blood samples were taken in all animals once a month by puncture of the caudal vein with new vacutainer equipment without anticoagulant. Samples were centrifuged at 3500 rpm for 10 min to obtain the serum that was then stored at −20 °C until its use. In cases of abortion, it was coming to perform necropsy fetus to obtain brain samples (20), in addition to collect a serum sample from the aborted cow, all samples were stored at −20 °C until its use; the age of the aborted fetus was calculated using the date of the last insemination of the cow. A sampling of the TMR supplied in the farm was conducted once a month, it was performed by means of the technique of “W” applied in the feeding of animals, so that took five samples of 1.0 kg, at a distance of 2 m about one another. The samples were placed in a new plastic bag, from which a sub-sample of 1.0 kg properly mixed was obtained stored at −20 °C until determination of ZEA (21).

### Serological test

The detection of total serum IgG specific to *N. caninum* was developed by applying the indirect ELISA test (19), using as antigen soluble extract of tachyzoites *N. caninum* obtained from cell culture at a concentration of 910 μg/ml, providing a concentration of 0.15 μg per well, diluted in carbonate-bicarbonate buffer (pH 9.6; 0.1M); sera were tested at a dilution of 1:100 in PBS-Tween 0.05%, using appropriate controls. The absorbance was measured at a wavelength of 405 nm. Serum samples were analyzed in duplicate and the average value of the optical density (OD) was converted into the relative index percent (IRPC) by using the following formula: IRPC = (O.D._405_ sample – O.D._405_ negative control) / (O.D._405_ positive control – O.D._405_ negative control) x100. Samples with IRPC≥ 8.2 were considered positive. The serostatus of the animals was investigated on a monthly basis throughout pregnancy or until due to abortion.

### Detection of DNA of N. caninum in aborted fetal tissue using nested PCR

Genomic DNA from brain tissue samples was extracted using a commercial Maxwell® 16 gDNA Purification Kit, developed for the automated Maxwell® 16 System (Promega, Wisconsin, USA), following the manufacturer’s instructions. For the detection of parasite DNA, a nested-PCR procedure based on the internal transcribed spacer 1 (ITS1) region of *N. caninum* was carried out (22). In each batch of PCR amplifications, DNA from *N. caninum* tachyzoites was included as positive controls. Negative controls, including reactions without template and extractions of bovine DNA negative to *N. caninum* by ITS-1 PCR, were also included at each batch of PCR’s. Secondary amplification product (249 bp) was visualized by 1.8% agarose gel electrophoresis and ethidium bromide staining (23).

### Detection of ZEA in food samples

The food samples were analyzed for the quantitative detection of ZEA through competitive ELISA, using a commercial kit (Ridascreen Fast Zearalenon SC, R-Biopharm AG, Darmstadt, Germany), having a detection limit of 5 μg/kg and for quantification 16 μg/kg. Prior to ELISA analysis, samples were milled to give a particle size between 500 and 800 μ, homogenized and instructions issued by the manufacturer were followed. The test was run with 50 μL of filtrate from the sample in duplicate, and reading the absorbance at 450 nm. The calculation of the results was performed according to the manufacturer’s instructions using the software provided by the same (RidaSoft Win).

### Detection of hormone levels in serum by radioimmunoassay (RIA)

The determination of the levels of E and P4 was developed monthly by solid phase RIA using the following packages: Coat-A-Count Progesterone and Coat-A-Count Estradiol (Siemens Healthcare Diagnostics, Inc., Los Angeles, CA.). The first with analytical sensitivity of 0.02 ng/ml, and an intra-assay coefficient of variation and inter 9.51 and 8.96%, respectively, and the second with analytical sensitivity of 8 pg/ml, with an intra-assay coefficient of variation and inter 5.92% and 6.0%, respectively. The test was developed in the Laboratory of Endocrinology, Department of Reproduction, Faculty of Veterinary Medicine and Animal Husbandry of the National Autonomous University of Mexico.

### Information analysis

The dynamic of seroestatus to *N. caninum* was described in all animals along the gestational period using average values of IRPC; these values were analysed using ANOVA and Student’s-T-test (*P*<0.05). The monthly level of ZEA in TMR was registered and the mean and standard deviation were calculated. Information on the results of the detection levels of E and P4 in serum underwent the procedure GEE (Generalized Estimating Equations), in order to detect differences between the studied variables (levels of E and P4) in the time (nine months pregnant) and its possible interaction with natural exposure to ZEA intake (*P*<0.05). The procedure was developed using Stata 13 statistical package (Stata Corp. LP). The following reproductive parameters were calculated: services per conception, pregnancy rate to first, second and third service, percentage of abortions and calving rate in each group under study.

The Committee on Use and Care of Animals of the Instituto Tecnológico El Llano Aguascalientes approved this project. The owner of the dairy farm gave their consent to study their animals. Adequate veterinary care was provided to all animals under study.

## Results

**Fig. 1:**
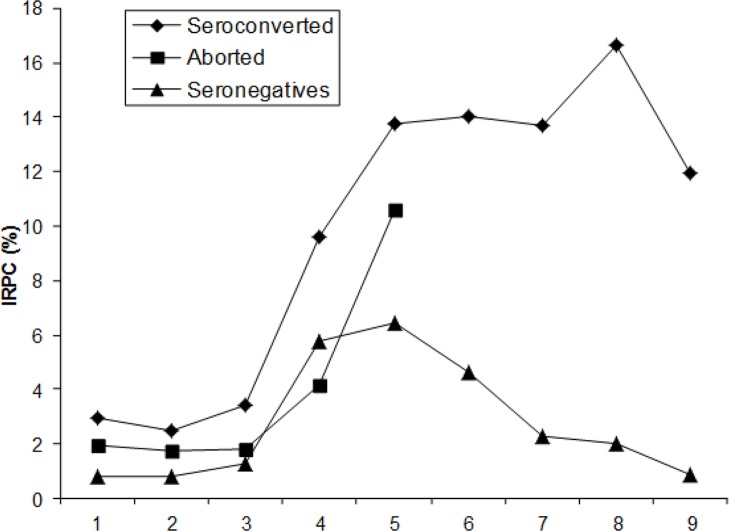
Dynamics of immune response (expressed as IRPC) during gestation of naturally infected heifers (Group A)

The dynamics of the immune response in the group A (seronegative) shown in Fig. 1. Three different subgroups were described: a) seronegative, in which 12/20 animals (60%) maintained their serostatus, b) seroconverted, in which 8/20 animals (40%) presented sero-status change, which happened in the second trimester, and c) aborted, with 3/8 animals which seroconverted before the abortion. The samples of fetal brain tissue analyzed by nested PCR detected DNA of *N. caninum* in all cases.

**Fig. 2:**
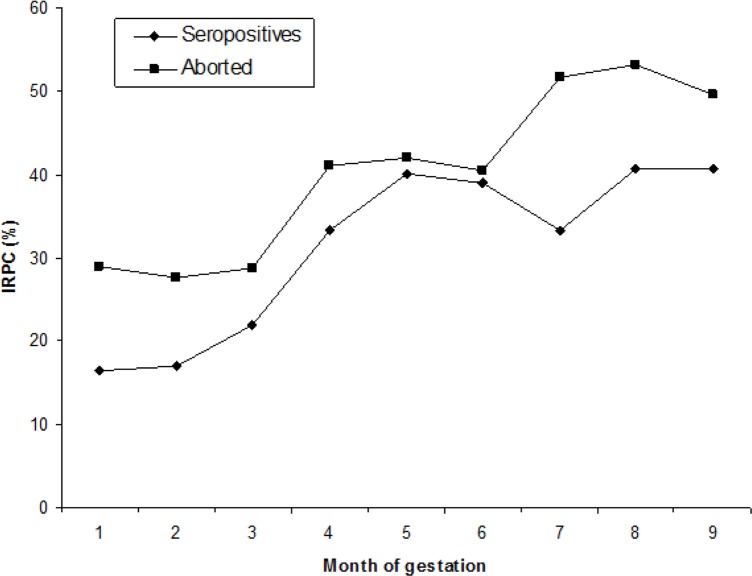
Dynamics of immune response (expressed as IRPC) during gestation of naturally infected heifers (Group B)

In the group B (seropositive), all animals maintained their serostatus, but two subgroups were described: a) seropositive, with 17/20, animals (85%) and b) aborted, with 3/20 animals (15%) that did in the last third of gestation (Fig. 2). The dynamics of the immune response in subgroups showed a similar pattern throughout gestation, detecting an increase in the IRPC from the third month that reached the highest level in the second third of gestation remaining at high levels until the end of it or at presentation of abortion. IRPC differences between the months of gestation were found (*P* <0.05). The samples of fetal brain tissue analyzed by nested PCR detected DNA of *N. caninum* in all cases.

The reproductive parameters of heifers are shown in Table 1, which were similar in the groups under study. In group A, two of the aborted fetuses had 120 d of gestational age and the other 150 d, while in group B, had 180, 210 and 240 d of gestation. No alterations were observed in reproductive health of animals. The presence of ZEA was detected throughout the study period; food samples analyzed showed levels in a range of 212 to 672 μg/kg, with a mean of 426 + 113.3 μg/kg as shown in the Table 2.

**Table 1:** Reproductive parameters of Holstein heifers under study

***Reproductive Parameter***	***Group A***	***Group B***
Services/Conception	1.7	1.5
Pregnancy rate (%) 1^st^ service	50	60
Pregnancy rate (%) 2^nd^ service	80	70
Pregnancy rate (%) 3^rd^ service	100	100
Abortions rate (%)	15	15
Calving rate (%)	85	85

**Table 2:** Monthly level of ZEA (μg/kg), determined in the TMR supplied to heifers under study.

***Month***	***1***	***2***	***3***	***4***	***5***	***6***	***7***	***8***	***9***	***10***	***11***	***Mean***	***± SD***
ZEA	672	212	375	400	450	383	535	400	389	400	470	426	113.3

**Fig. 3:**
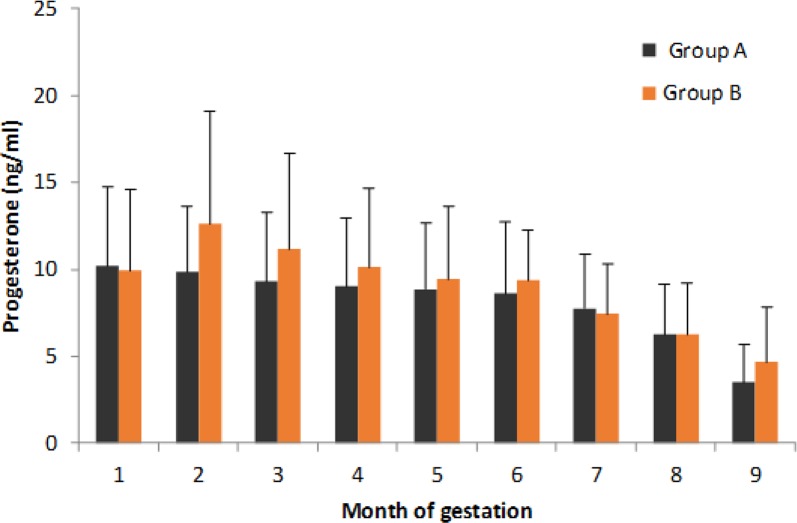
Mean Progesterone levels during gestation in groups under study

**Fig. 4:**
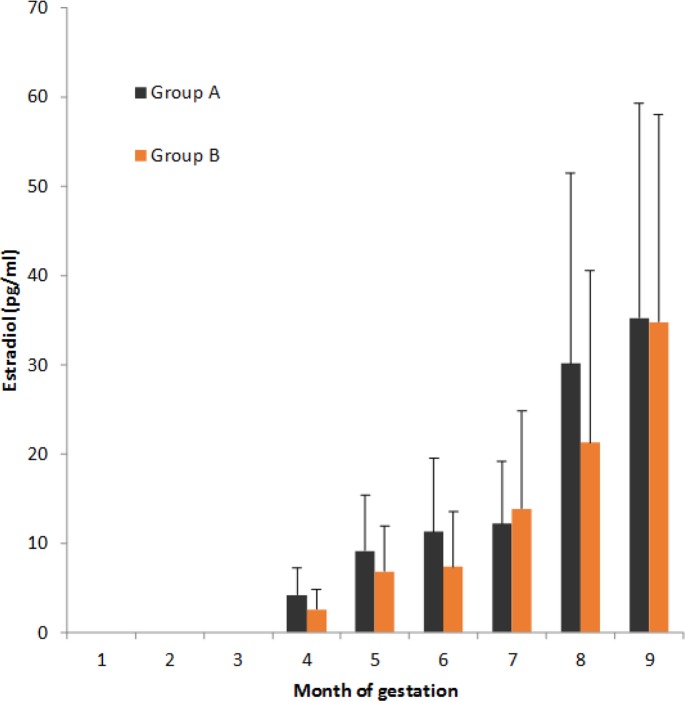
Mean Estradiol levels during gestation in groups under study

During pregnancy in animals that did not abort the presence of P4, both seropositive and seronegative, was evident from the beginning of the same, showing a decrease in the level of detection from the eighth months (Fig. 3). While for E, in both groups, the presence was undetectable or low in the first month and as the gestation progresses its value was increasing, reaching its highest value in the ninth month (Fig. 4). In the heifers that aborted the estradiol and P4 concentration was similar to those, they did not aborted. In all cases, in the sampling after the abortion was not possible to detect the presence of either hormone.

No statistical differences among the studied variables (levels of estradiol and progesterone) in time (nine months of gestation) were detected. It was not identified any interaction with the natural exposure to ZEA intake in any of the groups under study.

## Discussion

The dynamics of specific immune response to *N. caninum* recorded during pregnancy allows estimating that in group A, the high seroconversion maybe explains by the coexistence of horizontal transmission and reactivation of chronic infection. The first one there were five dogs on the farm that circulated freely in the facilities, and the second seronegative animals with parasite cysts in the brain which probably caused the seroconversion (24); other studies report that seronegative animals may have parasitemia, which could lead to seroconversion (8).

The mechanisms of transmission and perpetuation of *N. caninum* in a herd is diverse, and that endogenous transmission plays an important role that can sometimes be underestimated, while horizontal transmission, particularly in farms with the presence of the definitive host, is constant and with high efficiency (1, 25). The seropositive animals (group B), suffered a chronic infection, in consequence, showed high levels of total IgG during gestation, which initiated with an increase since the third month; chronic infection does not necessarily cause the abortion because most infected animals have normal pregnancies, however, they are able to efficiently transmit the infection to their offspring (1, 2, 25).

Different studies have documented an increase in the level of antibodies from the third month of gestation and may be more evident on the second third of gestation (26–31). In the present study, the third month is a starting point to experiment an increase in the level of total IgG. However, high records were detected in the second or third trimester of pregnancy, this behavior may be due to different factors internal and external related to the fetus and mother, which combine to define the destination of infection, either abortion or offspring apparently healthy but infected congenitally (25, 27). The heifers that had abortions in the group A shown an increase of total IgG before to abortion, since the third month and may be infected by horizontal transmission, in these cases the aborted fetuses had between 120 and 150 d of gestation. The aborted animals in the group B, had seropositive animals chronically infected because they showed an increase in the values of IRPC from the third month of gestation, aborted fetuses in this group had between 180 and 240 d of gestation. The frequency of abortions by *N. caninum* is greater between 150 and 210 d of gestation and that high levels of total IgG show the existence of a chronic infection (1, 13, 25). This observation confirms the wide parasite’s ability to remain in a herd.

The TMR supplied to heifers under study was contaminated naturally during the whole period of study with ZEA; the tolerable intake of ZEA in dairy cattle has not been clearly defined, but several authors believe that this value is 250 μg/kg, although others mention that the limit is up to 500 μg/kg (17, 32). Clinical signs of hyperestrogenism due ZEA intake are observed infrequently in cattle, and only following the ingestion of highly contaminated silage or a long-term exposure to contaminated feed materials (33, 34). Within the rumen, the protozoal population has the highest capacity to detoxify ingested mycotoxins (35). We expected an elevation in estradiol levels during gestation due long-term ingestion of ZEA, perhaps more important in *N. caninum* seropositive heifers and eventually in cases of abortion. However, the hormonal profile of heifers during gestation showed no abnormalities that indicate the presence of hyperestrogenism, even in seropositive or aborted animals. Therefore, no effect of ZEA is presented in the secretion of E and consequently in their relationship with P4, since the levels recorded in both hormones were observed within values reported in the literature as normal (36–39).

*N. caninum* modify endocrine patterns of P4, cortisol and PAG 2 (Pregnancy-Associated Glycoprotein) in animals chronically infected (40–42). *N. caninum* seropositive animals show concentrations of P4 lower than those identified in seronegative cows in the first six months of gestation (43). In the present study, no significant differences in the levels of P4 among seropositive or seronegative groups were observed during gestation. In animals under study, reproductive parameters not indicated evidence of infertility as might have been expected.

## Conclusion

The parasite has a wide capacity to perpetuate the infection in the herd and the chronic ingestion of ZEA through the TMR naturally contaminated with this mycotoxin does not affect serum concentrations of E and P4 during gestation of heifers seropositive and seronegative to *N. caninum* infection, and cannot be related with the infection for *N. caninum* and the abortion.

## References

[1] Dubey JP Schares G Neosporosis in animals-the last five years. Vet Parasitol. 2011;180(1–2):90–108. 21704458 10.1016/j.vetpar.2011.05.031

[2] Goodswen SJ Kennedy PJ Ellis JT A review of the infection, genetics, and evolution of *Neospora caninum*: From the past to the present. Infect Genet Evol. 2013;13:133–50. 22985682 10.1016/j.meegid.2012.08.012

[3] Almería S López-Gatius F Bovine neosporosis: Clinical and practical aspects. Res Vet Sci. 2013;95(2):303–9. 23659742 10.1016/j.rvsc.2013.04.008

[4] Reichel MP Alejandra Ayanegui-Alcérreca M Gondim LF What is the global economic impact of *Neospora caninum* in cattle - The billion dollar question. I Int J Parasitol. 2013;43(2):133–42. 23246675 10.1016/j.ijpara.2012.10.022

[5] Garcia-Vazquez Z Rosario-Cruz R Ramos-Aragon A *Neospora caninum* seropositivity and association with abortions in dairy cows in Mexico. Vet Parasitol. 2005;134(1–2):61–5. 16098675 10.1016/j.vetpar.2005.07.007

[6] Garcia-Vazquez Z Rosario-Cruz R Mejia-Estrada F Seroprevalence of *Neospora caninum* antibodies in beef cattle in three southern states of Mexico. Trop Anim Health Prod. 2009;41(5):749–53. 19016341 10.1007/s11250-008-9247-x

[7] Gutiérrez GJ Cruz-Vázquez C Medina-Esparza L Factores de manejo asociados con la seroprevalencia a la infección por *Neospora caninum* en ganado lechero de Aguascalientes, México. Vet Mex. 2007;38:261–270.

[8] Castañeda-Hernández A Cruz-Vázquez C Medina-Esparza L *Neospora caninum*: seroprevalence and DNA detection in blood of sheep from Aguascalientes, Mexico. Small Rumin Res. 2014;119:182–186.

[9] Sánchez GF Morales SE Martínez MJ Determination and correlation of anti-*Neospora caninum* antibodies in dogs and cattle from Mexico. Can J Vet Res. 2003;67(2):142–5. 12760481 PMC227043

[10] Cruz-Vázquez C Medina-Esparza L Marentes A Seroepidemiological study of *Neospora caninum* infection on dogs found in dairy farms and urban areas of Aguascalientes, Mexico. Vet Parasitol. 2008;157(1–2):139–43. 18722716 10.1016/j.vetpar.2008.07.004

[11] Olamendi-Portugal M Caballero-Ortega H Correa D Serosurvey of antibodies against *Toxoplasma gondii* and *Neospora caninum* in white-tailed deer from northern Mexico. Vet Parasitol. 2012;189(2–4):369–73. 22633992 10.1016/j.vetpar.2012.04.011

[12] Medina-Esparza L Macías L Ramos-Parra M Frequency of infection by *Neospora caninum* in wild rodents associated with dairy farms in Aguascalientes, Mexico. Vet Parasitol. 2013;191(1–2):11–4. 22989953 10.1016/j.vetpar.2012.08.007

[13] Dubey JP Schares G Ortega-Mora LM Epidemiology and control of neosporosis and *Neospora caninum*. Clin Microbiol Rev. 2007;20(2):323–67. 17428888 10.1128/CMR.00031-06 PMC1865591

[14] Bartels CJ Wouda W Schukken YH Risk factors for *Neospora caninum*-associated abortion storms in dairy herds in The Netherlands (1995 to 1997). Theriogenology. 1999;52(2):247–57. 10734392 10.1016/S0093-691X(99)00126-0

[15] Conzuelo SR Medina-Esparza L Ramos-Para M Factores de riesgo asociados a la seroprevalencia de anticuerpos a *Neospora caninum* en ganado lechero de Aguascalientes, México. Rev Mex Cienc Pec. 2011;2:15–24.

[16] Zinedine A Soriano JM Moltó JC Review on the toxicity, occurrence, metabolism, detoxification, regulations and intake of zearalenone: An oestrogenic mycotoxin. Food Chem Toxicol. 2007;45:1–18. 17045381 10.1016/j.fct.2006.07.030

[17] Cortinovis C Pizzo F Spicer LJ *Fusarium* mycotoxins: Effects on reproductive function in domestic animals: A review. Theriogenology. 2013;80(6):557–64. 23916251 10.1016/j.theriogenology.2013.06.018

[18] Vitela MI Cruz-Vázquez C Solano J Comportamiento de vacas Holstein mantenidas en un sistema de estabulación libre, en invierno, en zona árida, México. Arch Med Vet. 2005;37:23–27.

[19] Alvarez-García G Pereira-Bueno J Gómez-Bautista M Pattern of recognition of *Neospora caninum* tachyzoite antigens by naturally infected pregnant cattle and aborted foetuses. Vet Parasitol. 2002;107(1–2):15–27. 12072210 10.1016/s0304-4017(02)00091-2

[20] Medina L Cruz-Vázquez C Quezada T Survey of *Neospora caninum* infection by nested PCR in aborted fetuses from dairy farms in Aguascalientes, Mexico. Vet Parasitol. 2006;136(3–4):187–91. 16332413 10.1016/j.vetpar.2005.11.003

[21] Bautista A Santos S Manual de técnicas de muestreo para manejadores de recursos naturales. UNAM: México; 2004 p. 351–55.

[22] Buxton D Maley SW Wright S The pathogenesis of experimental neosporosis in pregnant sheep. J Comp Pathol. 1998;118(4):267–79. 9651804 10.1016/s0021-9975(07)80003-x

[23] Regidor-Cerrillo J Gómez-Bautista M Pereira-Bueno J Isolation and genetic characterization of *Neospora caninum* from asymptomatic calves in Spain. Parasitology. 2008;135(14):1651–9. 18980700 10.1017/S003118200800509X

[24] Santos SL de Souza Costa K Gondim LQ Investigation of *Neospora caninum*, *Hammondia* sp., and *Toxoplasma gondii* in tissues from slaughtered beef cattle in Bahia, Brazil. Parasitol Res. 2010;106(2):457–61. 19943064 10.1007/s00436-009-1686-4

[25] Dubey JP Buxton D Wouda W Pathogenesis of bovine neosporosis. J Comp Pathol. 2006;134:267–289. 16712863 10.1016/j.jcpa.2005.11.004

[26] Stenlund S Kindahl H Magnusson U Serum antibody profile and reproductive performance during two consecutive pregnancies of cows naturally infected with *Neospora caninum*. Vet Parasitol. 1999;85(4):227–34. 10488725 10.1016/s0304-4017(99)00120-x

[27] Pereira-Bueno J Quintanilla-Gozalo A Seijas-Carbalfedo A Observational studies in *Neospora caninum* infected dairy cattle: pattern of transmission and age-related antibody fluctuations. In: (Eds. A. Hemphill; B. Gottstein). A European perspective on *Neospora caninum*. Int J Parasitol. 2000;30:906–909.

[28] Guy CS Williams DJL Kelly DF *Neospora caninum* in persistently infected pregnant cows: spontaneous transplacental infection is associated with an acute increase in maternal antibody. Vet Rec. 2001;149(15):443–9. 11688746 10.1136/vr.149.15.443

[29] Piergili Fioretti D Pasquali P Diaferia M *Neospora caninum* infection and congenital transmission: serological and parasitological study of cows up to the fourth gestation. J Vet Med B Infect Dis Vet Public Health. 2003;50(8):399–404. 14633211 10.1046/j.1439-0450.2003.00686.x

[30] Andrianarivo AG Anderson ML Rowe JD Immune responses during pregnancy in heifers naturally infected with *Neospora caninum* with and without immunization. Parasitol Res. 2005;96(1):24–31. 15841416 10.1007/s00436-005-1313-y

[31] Špilovská S Moskwa B Reiterová K Kinetics of anti-*Neospora* antibodies during the period of two consecutive pregnancies in chronically infected dairy cows. Acta Parasitol. 2013;58(4):463–7. 24338306 10.2478/s11686-013-0174-2

[32] Minervini F Dell’Aquila ME Zearalenone and reproductive function in farm animals. Int J Mol Sci. 2008;9(12):2570–84. 19330093 10.3390/ijms9122570 PMC2635636

[33] Bloomquist C Davidson JN Pearson EG Zearalenone toxicosis in prepubertal dairy heifers. J Am Vet Med Assoc. 1982;180(2):164–5. 6460728

[34] Coppock RW Mostrom MS Sparling CG Apparent zearalenone intoxication in a dairy herd from feeding spoiled acid-treated corn. Vet Hum Toxicol. 1990;32(3):246–8. 2141202

[35] Fink-Gremmels J The role of mycotoxins in the health and performance of dairy cows. Vet J. 2008;176(1):84–92. 18342554 10.1016/j.tvjl.2007.12.034

[36] Pope GS Gupta SK Munro IB Progesterone levels in the systemic plasma of pregnant, cycling and ovariectomized cows. J Reprod Fertil. 1969;20(3):369–81. 5390876 10.1530/jrf.0.0200369

[37] Knickerbrocker JJ Drost M Tatcher WW Endocrine patterns during the initiation of puberty, the estrous cycle, pregnancy and parturition in cattle. In: Morrow DA, editor. Current Theraphy in Theriogenology 2. Saunders: Philadephia 1986 p. 117–25.

[38] Pape-Zambito DA Magliaro AL Kensinger RS 17Beta-estradiol and estrone concentrations in plasma and milk during bovine pregnancy. J Dairy Sci. 2008;91(1):127–35. 18096933 10.3168/jds.2007-0481

[39] Franco J Uribe VLF Hormonas reproductivas de importancia veterinaria en hembras domésticas rumiantes. Biosalud. 2012;11:41–56.

[40] Abdelfatah-Hassan A Almería S Serrano B The inseminating bull and plasma pregnancy-associated glycoprotein (PAG) levels were related to peripheral leukocyte counts during the late pregnancy/early postpartum period in high-producing dairy cows. Theriogenology. 2012;77(7):1390–7. 22225694 10.1016/j.theriogenology.2011.11.002

[41] García-Ispierto I Nogareda C Yániz JL *Neospora caninum* and *Coxiella burnetii* seropositivity are related to endocrine pattern changes during gestation in lactating dairy cows. Theriogenology. 2010;74(2):212–20. 20416940 10.1016/j.theriogenology.2010.02.004

[42] García-Ispierto I Almería S Serrano B Plasma concentrations of pregnancy-associated glycoproteins measured using anti-bovine PAG-2 antibodies on day 120 of gestation predict abortion in dairy cows naturally infected with *Neospora caninum*. Reprod Domest Anim. 2013;48(4):613–8. 23228018 10.1111/rda.12134

[43] García-Ispierto I Serrano-Pérez B Almería S Effects of crossbreeding on endocrine patterns determined in pregnant beef/dairy cows naturally infected with *Neospora caninum*. Theriogenology. 2015;83(4):491–6. 25459029 10.1016/j.theriogenology.2014.10.013

